# Investigation of Surface Acoustic Wave Propagation Characteristics in New Multilayer Structure: SiO_2_/IDT/LiNbO_3_/Diamond/Si

**DOI:** 10.3390/mi12111286

**Published:** 2021-10-21

**Authors:** Hanqiang Zhang, Hongliang Wang

**Affiliations:** National Key Laboratory for Electronic Measurement Technology, Key Laboratory of Instrumentation Science and Dynamic Measurement, Ministry of Education, North University of China, Taiyuan 030051, China; s1906104@st.nuc.edu.cn

**Keywords:** surface acoustic wave (SAW), temperature compensation, layered structure, finite element method (FEM), filter

## Abstract

Surface acoustic wave (SAW) devices are widely used in many fields such as mobile communication, phased array radar, and wireless passive sensor systems. With the upgrade of mobile networks, the requirements for the performance of SAW devices have also increased, and high-frequency wideband SAW devices have become an important research topic in communication systems and other application fields. In this paper, a theoretical study for the realization of a layered SAW filter based on a new SiO_2_/IDT/128°YX-LiNbO_3_/diamond/silicon layered structure using the modeling software COMSOL Multiphysics is presented. The effects of lithium niobate (LiNbO_3_), an interdigital transducer (IDT), and SiO_2_ thin films on the evolution of the phase velocity, electromechanical coupling coefficient (k^2^), and temperature coefficient of frequency were studied by employing a finite element method simulation. Furthermore, a longitudinal coupling resonator filter was designed. To investigate the SAW characteristics of the filter, a transient analysis was conducted to calculate the electrical potential and particle displacement under the resonance condition and to analyze the frequency response. The study concluded that this new multilayer structure can be applied to design and manufacture a variety of high-frequency and wideband SAW filters with a temperature compensation function, for operation above the GHz range.

## 1. Introduction

Surface acoustic wave (SAW) devices are widely used in mobile communication, industrial control, and environmental and biological sensors because of their good consistency, small size, strong anti-interference ability, and low cost. With the progression of high-frequency and broadband communication, the development of high-frequency devices such as SAW devices and film bulk acoustic resonators used in the GHz range has become an emerging research hotspot [1]. For the dissemination of the fifth-generation mobile communication system (5G) and the development of next-generation communication systems, SAW devices with performances higher than those of the existing 4G communication system equipment, while having a higher frequency, larger electromechanical coupling coefficient ($k^{2}$), higher quality (Q) factor, and smaller temperature coefficient of frequency (TCF), are required [2]. Due to the increasing demand for large-volume data transmission and mobile communication, devices operating at high frequencies are required. The working frequency of a SAW device is mainly determined by the finger width of the interdigital structure and the velocity of the substrate material. Thus, there are two main ways to increase the working frequency [3]: (1) reduce the finger width of the interdigital transducer, which is limited by the photolithography resolution, and the finer interdigital electrode greatly increases the process difficulty and ohmic loss; (2) use high-phase-velocity piezoelectric materials, but general single-crystal materials such as lithium niobate (LiNbO_3_ and LNO) and zinc oxide (ZnO) have low sound velocity, and even some doped piezoelectric materials cannot significantly improve the phase velocity. However, the new multilayer structure can solve this problem as the complementary advantages between materials can be utilized by selecting a multilayer medium comprising different materials. For example, the surface source or interface source structure created by the deposition of a piezoelectric film on some high-speed materials can improve the velocity of the SAW and the electromechanical coupling coefficient simultaneously. The multilayer structure has the advantages of not only high sound speed, large electromechanical coupling coefficient, and better frequency and temperature stability but also process simplicity and economic cost savings, which reduces the requirements of photolithography technology in the production of high-frequency devices.

Because of the unique advantages of multilayer structures, many scholars have conducted extensive and in-depth research on multilayer structures in recent years. S. Maouhoub [4] investigated the propagation characteristics of SAWs in ZnO/Si and ZnO/AlN/Si structures, and numerically analyzed the phase velocity, coupling coefficient, and temperature coefficient of the frequencies of the first two modes of SAWs for the ZnO/Si structure and compared them with experimental data. L. Wang [5] fabricated SAW devices using embedded interdigital transducers (IDTs) on an AlN/diamond/Si layered substrate, and their performances were investigated. The Sezawa mode is the dominant resonance mode with the highest resonant frequency of up to 17.7 GHz, a signal amplitude of 20 dB, and an electromechanical coupling coefficient of 0.92%. Comparing these SAW devices with those having conventional IDTs on the same layered structure, the output SAW power and resonant frequency of the devices are improved by 10.7% and 1.1%, respectively. B. Xie [6] described an innovative electroacoustic coupling structure with a patterned piezoelectric film as an IDT on a diamond substrate for SAW devices with better quality factor and frequency resolution. Y. Wang [7] studied the propagation characteristics of SAWs in a ZnO film/R-sapphire structure. It was found that the first Rayleigh wave has an exceedingly large $k^{2}$ value of 4.95% in the ZnO film/R-sapphire substrate, associated with a phase velocity of 5300 m/s.

Because the diamond has an extremely high elastic modulus (*E* = 1200 GPa), a high longitudinal wave sound velocity (18 km/s), and a low material density (*ρ* = 3.51 g/cm^3^), it can be combined with a piezoelectric film to prepare high-frequency SAW devices with unique advantages. Further, because of its excellent thermal conductivity, the diamond SAW device has the ability to communicate with super-high power, even at high frequencies, and its performance can exceed that of traditional SAW devices. However, because of the lack of piezoelectric properties, it is necessary to add piezoelectric thin-film layers. LiNbO_3_ crystals are multifunctional materials with favorable piezoelectric, nonlinear optical and electro-optic properties and can complement the advantages of diamond substrates to produce SAW devices with a layered structure. Although LiNbO_3_ exhibits the highest piezoelectricity among piezoelectric materials, it has poor temperature stability. Meanwhile, SiO_2_ has excellent temperature stability; the authors have designed a new SAW substrate with a large coupling factor and excellent temperature stability by combining LiNbO_3_ and SiO_2_ films. Moreover, there are multiple modes of SAWs in multilayered structures. High-order SAWs or bulk acoustic waves with faster propagation speeds can also be used in the design, and the best mode of surface waves can be selected for different design requirements.

In this study, to solve the problems of low working frequency, narrow bandwidth, and poor temperature stability of traditional single-crystal structure SAW devices, a new longitudinally coupled SAW resonator filter (LCRF) with SiO_2_/IDT/128°YX-LiNbO_3_/diamond/Si layered structure was designed. Because the propagation characteristics of SAWs are affected by the film thickness of each layer, an accurate calculation of the SAW characteristics under different film thicknesses is the theoretical basis for the design and fabrication of high-performance SAW filters using this composite structure. In this work, the vibration mode and admittance characteristics of two-dimensional (2D) element structures were obtained by changing the thickness of each film layer, and the propagation characteristics of Rayleigh and Sezawa waves were accurately analyzed. The propagation state of the acoustic signal in the 2D complete structure was obtained via time-domain analysis. The results show that the structure can have good application prospects in the design of high-frequency and wideband SAW devices (such as filters and duplexers) with temperature compensation functions in the future.

## 2. Simulation Methodology and Device Structure

### 2.1. Simulation Methodology

Piezoelectric effect is a phenomenon of mutual conversion between mechanical energy and electrical energy, which involves the interaction between electrical quantity and mechanical quantity. In this paper, COMSOL Multiphysics 5.6 is used to simulate and analyze the structure. In COMSOL, the coupling between structure domain and electrical domain can be expressed as the relationship between material stress and dielectric constant under constant stress, or the coupling between material strain and dielectric constant under constant strain [8]. Finite Element Method (FEM) couples between the stress (*T*), strain (*S*), electric field (*E*), and electric displacement (*D*) in a stress-charge of a piezoelectric crystal. This coupling is governed by the piezoelectric constructive equations [9] given by:

(1) $$T_{ij}=c_{ijkl}^{E}S_{kl}-e_{kij}E_{k}$$

(2) $$D_{i}=e_{ijk}S_{jk}+\varepsilon_{ij}^{S}E_{j}$$

where, $T_{ij}$ represents the stress tensor, $c_{ijkl}^{E}$, $e_{ijk}$, and $\varepsilon_{ij}$ correspond to the material stiffness, coupling properties, and relative permittivity, $E_{k}$ is the electric field vector (V/m), $S_{kl}$ is the strain vector and $D_{i}$ is the electrical displacement (C/m^2^). The basic wave equation in the piezoelectric substrate can be derived mathematically from Newton’s equation and Maxwell’s equation, which couple the electric potential and displacement components together [10]:

(3) $$c_{ijkl}\frac{\partial^{2}u_{l}}{\partial x_{j}\partial x_{k}}+e_{kij}\frac{\partial^{2}\phi }{\partial x_{j}\partial x_{k}}=\rho \frac{\partial^{2}u_{i}}{\partial t^{2}}$$

(4) $$\varepsilon_{ij}\frac{\partial^{2}\phi }{\partial x_{i}\partial x_{j}}-e_{ijk}\frac{\partial^{2}u_{j}}{\partial x_{i}\partial x_{k}}=0$$

where ρ, $u_{l}$ and ϕ are, respectively, the mass density, particle displacement and scalar electric potential. Where i,j,k=1, 2, and 3.

### 2.2. Model Description

**Figure 1:**
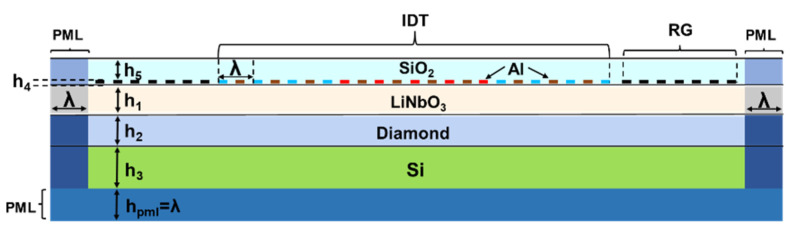
Schematic representation of the SiO_2_/IDT/LiNbO_3_/diamond/Si multilayer structure.

**Figure 2:**
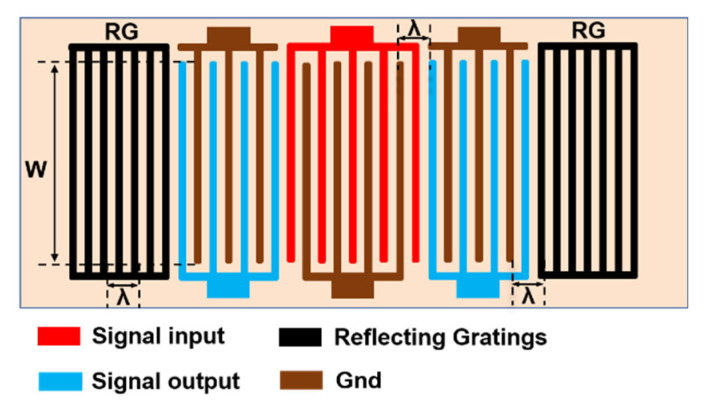
IDT design of the LCRF used in this study.

SiO_2_ thin film can be used as temperature compensation layer to achieve high temperature stability of SAW filter. Figure 1 displays a schematic representation of the SiO_2_/IDT/LiNbO_3_/diamond/Si structure considered in the modeling, and the structure of the LCRF in this design is shown in Figure 2. It is clear that the LCRF is suitable for obtaining SAW filters with a wide bandwidth and low insertion loss.

**Figure 3:**
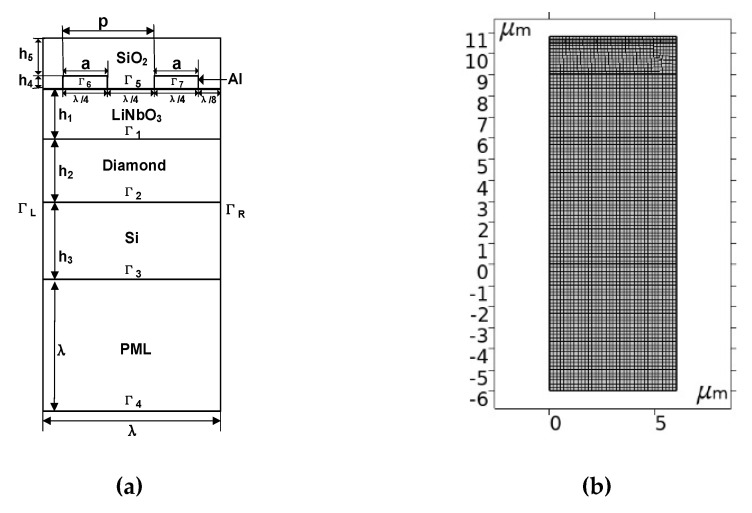
(**a**) Schematic representation of the studied structure in the model. (**b**) Single pair IDT model meshing, quadrilateral elements = 3675.

FEM simulation is an essential step in the design and fabrication of SAW devices. The device simulation can not only improve design accuracy, but it can also reduce unnecessary material losses. In this article, the software used for FEM analysis is COMSOL Multiphysics 5.6, which can easily perform piezoelectric coupling analysis and is used to analyze the response of 2D or 3D structural devices to changes in voltage and mechanical load. Thus, this software is suitable for the analysis and simulation of SAW devices. The simulation results can be used as the reference for the design and optimization of future SAW devices [11]. Because the interdigital electrodes of the IDT are periodically distributed, which reduces the simulation time, a simulation model can be established through one cycle. Considering that the Rayleigh wave and its higher-order mode vibrations are mainly limited to one wavelength (λ) on the surface, a 2D unit model with a substrate thickness greater than 1.5λ was established, as shown in Figure 3.

In Figure 3a, *p* is the distance between two adjacent metal electrodes; a is the width of the metal electrode; and $h_{1}$, $h_{2}$, $h_{3}$, $h_{4}$, and $h_{5}$ indicate the thicknesses of the lithium niobate, diamond, silicon, metal, and SiO_2_ layers, respectively. To eliminate the influence of the metallization rate and obtain the maximum reflectivity of the reflector, the metallization rate (*MR* = *a/p*) was designed to be 0.5. After the metallization rate was determined, because the propagation characteristics of the layered SAW structure are independent of the wavelength, the SAW wavelength was designed to be λ = 6 μm. Because most of the energy of the SAW is confined to the surface, and the thicknesses $h_{2}$ and $h_{3}$ are excluded, it was set to a fixed value while $h_{1}$, $h_{4}$, and $h_{5}$ were designed as variables. An aluminum electrode with a wavelength of 6 μm was deposited on the LiNbO_3_ surface. Silicon is assumed to be a semi-infinite substrate. The film thickness ratios $h_{1}/a$ and $h_{5}/a$ had ten values in the range of 0.1 to 1 with the interval of 0.1, and $h_{4}/a$ had five values in the range of 0.1 to 0.5, with the interval of 0.1. A perfectly matched layer (PML) can absorb propagating waves, accelerate the attenuation of evanescent waves, and perfectly match arbitrary lossy media [12]; therefore, a PML of 1λ was set in this study. In COMSOL, the model needs to be meshed. As shown in Figure 3b, to ensure calculation accuracy, we divided the minimum free quadrilateral unit, the smallest unit being 1.26 nm. For the IDT structure, *N_G_*, *N_IN_*, and *N_OUT_* indicate the number of reflective gratings, input fork index, and output fork index, respectively. The specific geometric parameters involved are shown in Table 1.

### 2.3. Material Parameters in the Model

The materials used in the finite element simulation included aluminum, lithium niobate, diamond, silicon dioxide, and silicon. The main characteristic parameters of materials [13,14,15,16,17,18,19,20] are shown in Table 2.

### 2.4. Setting of Boundary Conditions

In order to make the simulation results more accurate and reduce the amount of simulation calculations, the modal analysis of the layered LCRF is generally performed using a pair of interdigitated electrodes with periodic boundary conditions. The detailed electrical and mechanical boundary conditions of the numerical model are listed in Table 3.

## 3. Performance Impact Analysis

### 3.1. Modal Analysis

**Figure 4:**
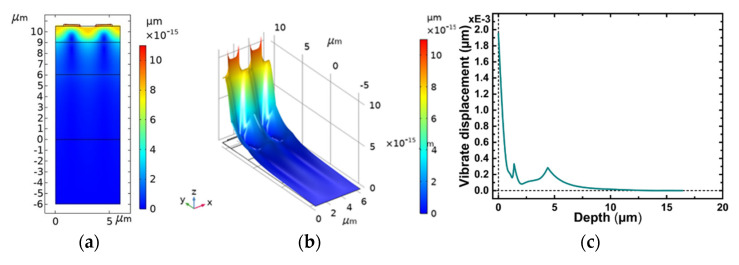
Plot of the displacement versus substrate depth of the proposed device at Rayleigh mode. (**a**) is a two-dimensional result and (**b**) is a three-dimensional result (**c**) is the calculated displacement of acoustic waves in depth direction.

The finite element analysis of the SiO_2_/IDT/LiNbO_3_/diamond/Si layered unit structure was carried out with COMSOL Multiphysics software, and the COMSOL Multiphysics model used in this work was built in 2D Structural Mechanics Module, with emphasis on the analysis of its resonance mode and frequency response. Through the modal analysis, symmetrical modes and antisymmetric modes can be obtained, and the occurrence of their resonant frequency $f_{\mathrm{sc}+}$ and anti-resonant frequency $f_{\mathrm{sc}-}$ is caused by the effect of metal electrode [21]. Plots of the longitudinal displacement versus substrate depth are shown in Figure 4a,b. The results indicate that the more it approaches the surface, the greater the vibration energy of the SAW, and the more obvious the vibration. Figure 4c clearly shows that the vibration intensity decreases exponentially as the depth of the substrate increases. It can be seen that most of the vibration energy during Rayleigh waves propagation on the substrate is within one wavelength, which is consistent with the research in the literature [22]. The results demonstrate the reliability of the model.

According to the working principle of the SAW filter, the SAW excited by the IDT can be regarded as the superposition of multiple pairs of IDT excitation signals. When the finger distance p is an integer multiple of the SAW half-wavelength λ/2, the IDT can superimpose the largest excitation signal. Therefore, the relationship between the intrinsic frequency of the SAW and *p* can be obtained as follows [23]:

(5) $$f_{0}=\frac{v_{p}}{2p}$$

where $v_{p}$ is the phase velocity of sound wave, $f_{0}$ is the central frequency of the SAW device. The propagation velocity $v_{p}$ of SAW can be given by:

(6) $$v_{p}=p\left(f_{\mathrm{sc}+}+f_{\mathrm{sc}-}\right)$$

The electromechanical coupling coefficient $k^{2}$ can be calculated by the following formula [24]:

(7) $$k^{2}=\frac{\pi^{2}(}{4}\frac{f_{\mathrm{sc}+}-f_{\mathrm{sc}-})}{f_{\mathrm{sc}+}}$$

The *TCF* is obtained by substituting the phase velocity calculated at temperature *T* and *T*_0_ = 25 °C for $v_{p}$ in the following equation:

(8) $$TCF=\frac{1}{T-T_{0}}\frac{v\left(T\right)-v\left(T_{0}\right)}{v\left(T_{0}\right)}$$

**Figure 5:**
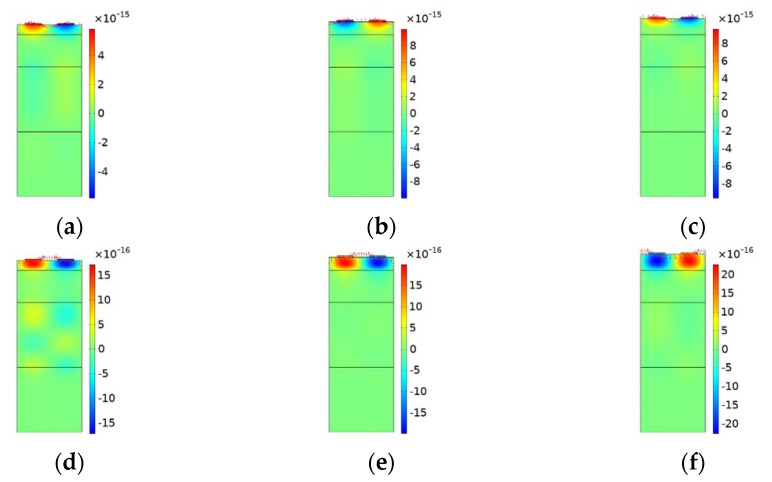
The acoustic wave modes shapes and their X-component of displacement (μm). (**a**–**c**) Anti-resonance of Rayleigh mode. (**d**–**f**) Anti-Resonance of Sezawa mode. (**a**,**d**) When $h_{1}/a=0.6$. (**b**,**e**) When $h_{1}/a=0.8$ and (**c**,**f**) when $h_{1}/a=1.0$.

Because the acoustic velocity of the diamond substrate is higher than that of the LiNbO_3_ piezoelectric film, multiple modes such as Rayleigh, Sezawa, and high-order Sezawa modes coexist in the same layered structure. First, we built the LiNbO_3_/diamond/Si model to precisely determine the acoustic wave propagation properties on this layered structure. The Rayleigh and Sezawa anti-symmetric resonant modes of the X-displacement component of the 2D unit structure when the piezoelectric film thickness ratio ($h_{1}/a$) is 0.6, 0.8, and 1.0, respectively, are presented in Figure 5. The results show that in response to the increase in LiNbO_3_ thickness, the Rayleigh mode exhibited the largest displacement due to particle displacement close to the free surface. As the order of the mode increased (Sezawa), the magnitude of the particle displacement decreased. The Rayleigh vibration mode had a larger amplitude than the Sezawa mode, and the amplitude declined with a depth quicker than that observed in the Sezawa mode because the Rayleigh wave energy is mainly concentrated on the surface, whereas the Sezawa wave energy is distributed in the base. In addition, as the thickness of the piezoelectric layer increased, the SAWs were increasingly confined near the surface.

### 3.2. Analysis of Surface Acoustic Wave Propagation Characteristics

The performance of a SAW device with a multilayer film structure is not only affected by the electrode structure of the transducer and the characteristics of the piezoelectric material, but also related to the thickness of the piezoelectric film and the thickness of the interdigital electrode. In the layered structure, the SAW is a dispersive wave, and the acoustic parameters are dispersive, as determined by the thickness ratio of the piezoelectric and other films to the wavelength of the acoustic wave. Therefore, setting appropriate geometric parameters can significantly improve the performance of SAW devices. To obtain the best performance structure, a detailed simulation analysis was performed on the thickness of the interdigital electrode, piezoelectric layer, and temperature compensation layer. First, we studied the effects of the metal electrode and piezoelectric film on the propagation characteristics of SAWs in the IDT/LiNbO_3_/diamond/Si structure and then investigated the influence of thickness variation of the piezoelectric layer and thermal compensating layer on the key performance parameters of the LCRF in the SiO_2_/IDT/LiNbO_3_/diamond/Si structure.

#### 3.2.1. Effects of Electrode Film Thickness on SAW Propagation Characteristics

**Figure 6:**
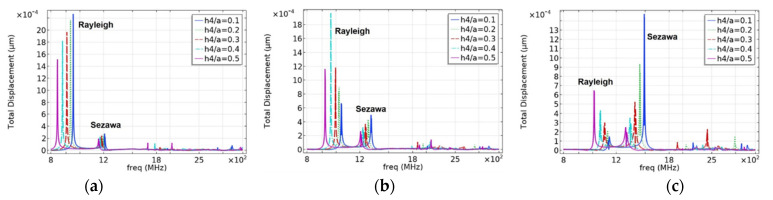
The effect of electrode thickness on resonant displacement. (**a**) When $h_{1}/a=1.0$. (**b**) When $h_{1}/a=0.8$ and (**c**) when $h_{1}/a=0.6$.

**Figure 7:**
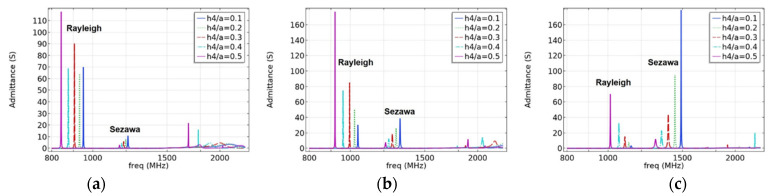
The effect of electrode thickness on admittance. (**a**) When $h_{1}/a=1.0$. (**b**) When $h_{1}/a=0.8$ and (**c**) when $h_{1}/a=0.6$.

**Figure 8:**
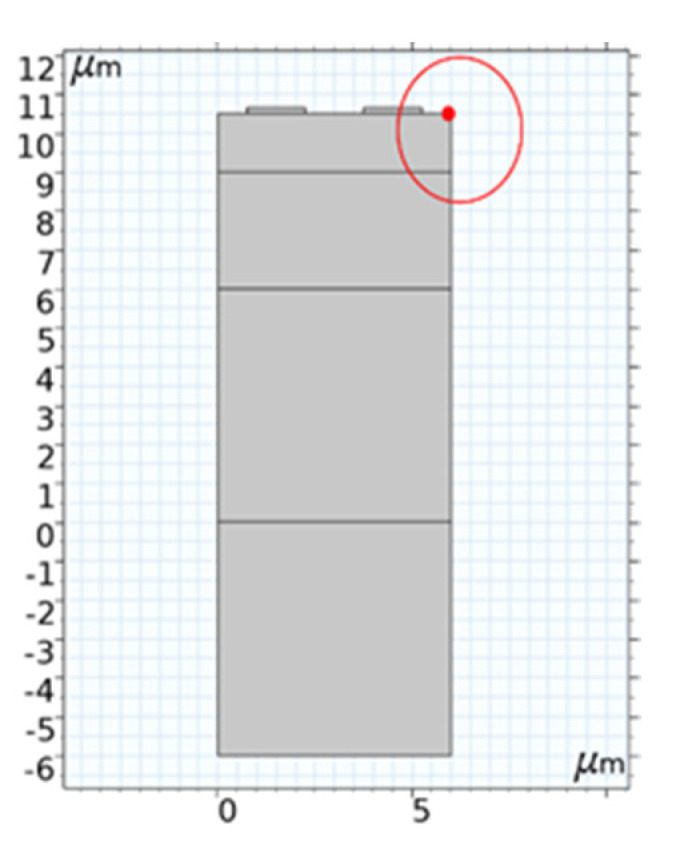
Position of total displacement probe.

The mass loading effect of the metal electrode will be introduced when the surface of piezoelectric medium is covered with metal film [25]. To study the implications of interdigital electrodes on the SAW propagation characteristics, the resonant displacement and admittance under different electrode film thickness ratios ($h_{4}/a$) were compared and analyzed, as presented in this section. In this design, the surface source (I-F) structure excitation was adopted, and the substrate was assumed to be a semi-infinite substrate. To eliminate the interference of other layer thicknesses, we set the values of $h_{1}/a$ to 0.6, 0.8, and 1; the other structural parameters are listed in Table 1. Figure 6 shows the simulation results of the frequency characteristics of different electrode thickness ratios $h_{4}/a$, and Figure 7 shows the effect of different electrode thicknesses on the admittance characteristics. It should be noted that the results in Figure 6 were probed at the red dot shown in Figure 8.

As shown in Figure 6a, as the electrode thickness decreases, the resonance frequency and displacement of the Rayleigh and Sezawa waves gradually increase, and the electrode mass loading effect is apparent. When $h_{4}/a=0.1$, that is, $h_{4}=0.15\;\mu m$, the resonance frequency and displacement reach their maximum values. In other cases, the resonance displacement in the Rayleigh wave decays as the electrode thickness decreases, as shown in Figure 6b,c. In contrast, as the thickness of the piezoelectric layer decreases, the influence of the electrode thickness on the Sezawa wave increases, and the Sezawa mode shows strict regularity. The variation rules of the results in Figure 7 are consistent with those in Figure 6. In either case, the resonance frequency decayed with an increase in the electrode thickness. There are other types of waves in the high-frequency range, but they were not considered in this study because of their small influence.

**Figure 9:**
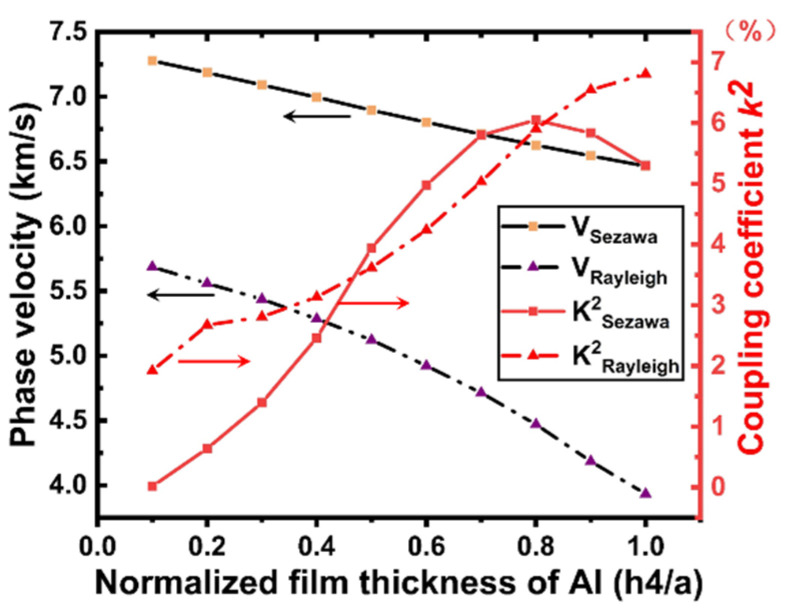
$v_{p}$ and $k^{2}$ dependence on $h_{4}/a$ (when $h_{1}/a\;$= 1).

For a unit model with fixed 1.5 μm LiNbO_3_ layer thickness, the phase velocities of the Rayleigh mode and Sezawa mode drop with increasing electrode thickness, while $k^{2}$ exhibits a rising trend (plotted in Figure 9).

As shown in Figure 9, the phase velocities decrease with the increasing electrode thickness for all wave modes. The phase velocity changes from 5686 to 3994 m/s for Rayleigh and from 7277 m/s to 6484 m/s for Sezawa. This indicates that the phase velocity of the Rayleigh wave decreases rapidly with an increase in the electrode thickness, whereas the phase velocity of the Sezawa wave is less affected by the electrode thickness. Moreover, this difference confirms that Rayleigh waves are concentrated on the surface of the piezoelectric thin film. In addition, it is clear that both the Rayleigh and Sezawa modes have a large $k^{2}$ value, exhibiting an upward trend for both. A high $k^{2}$ value (greater than 5.75%) was obtained for Sezawa in the $h_{4}/a$ range of 0.5–0.8, and the value peaked at $h_{4}/a$ = 0.8.

When the electrode thickness is zero or very small, the resonant and anti-resonant frequencies are basically the same, but with an increase in the electrode thickness, the Rayleigh wave acoustic field begins to move to the surface of the piezoelectric layer; the resonant and anti-resonant frequencies become significantly different and, according to Formula (7), correlate with the corresponding $k^{2}$. Simultaneously, the acoustic field of the Sezawa wave is mainly distributed at the interface between the piezoelectric film and the substrate; therefore, the mass loading effect of the electrode has a limited contribution to the velocity of the Sezawa wave. As the electrode thickness increased, the sound field began to move toward the surface of the piezoelectric layer. In this process, the distributions of the sound field and the electric field begin to match, and when the electrode thickness reaches a certain value, $k^{2}$ can reach the maximum value. As the thickness of the electrode continued to increase, the acoustic energy began to concentrate inside the electrode. At this time, the acoustic and electric fields begin to mismatch, and the coupling coefficient begins to decrease. Comparing the results presented in Figure 6, Figure 7 and Figure 9, when $h_{4}/a\;$= 0.1, it can be observed that these SAWs have the highest sound velocity, a good coupling coefficient, and significant admittance characteristics. Therefore, this electrode parameter was used in the subsequent studies.

#### 3.2.2. Effects of Piezoelectric Layer Thickness on SAW Propagation Characteristics

The characteristics of SAWs in piezoelectric multilayer media are not only affected by the characteristics of the piezoelectric layer material but also depend on the thickness of the piezoelectric layer. Initially, to validate our simulation method for the SiO_2_/IDT/LiNbO_3_/diamond/Si structure, we first used the IDT/LiNbO_3_/diamond/Si structure. In the next stage of the study, a thin film of SiO_2_ was coated over the surface of the LiNbO_3_ film, and the resonance frequencies of both modes with different thicknesses of the LiNbO_3_ film were recorded. The other structural parameters are listed in Table 1.

**Figure 10:**
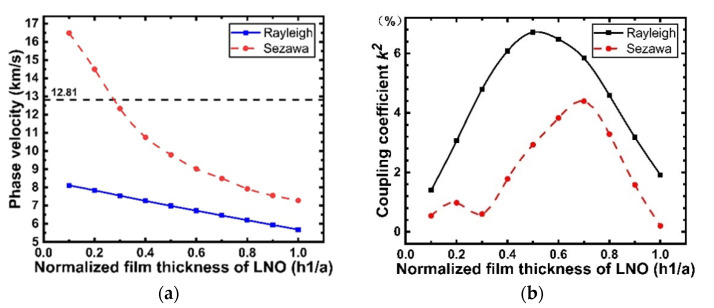
(**a**) SAW velocity versus normalized thickness of LiNbO_3_ in SiO_2_-free structure; (**b**) Electromechanical coupling coefficient versus normalized LiNbO_3_ thickness in SiO_2_-free structure.

The simulation results for the propagation characteristics of the IDT/LiNbO_3_/diamond/Si structure are presented in Figure 10. The phase velocity of the Rayleigh mode decreases with increasing normalized LiNbO_3_ film thickness, whereas that of the Sezawa mode decreases more dramatically. As displayed in Figure 10a, the value of the phase velocity changes from 8110 to 5660 m/s in the Rayleigh mode, and the value of the phase velocity changes from 16.48 to 7.28 km/s. When $h_{1}/a$ is less than 0.3, the Sezawa wave velocity is larger than that of the diamond shear wave (12.81 km/s). Meanwhile, the Sezawa wave will leak energy to the diamond substrate, thus increasing the insertion loss. As shown in Figure 10b, for the Rayleigh wave, $k^{2}$ first increases with an increase in $h_{1}/a$, reaches the maximum value of 6.71% when $h_{1}/a$ = 0.5, and then starts to decrease with a further increase in $h_{1}/a$.

**Figure 11:**
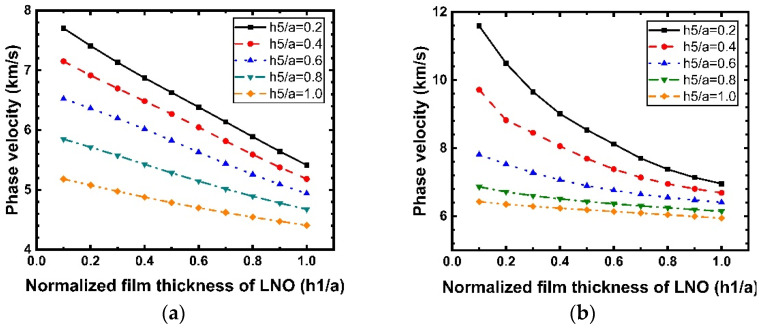
Dispersion curves of the Rayleigh and Sezawa waves velocity calculated for various values of $h_{1}/a$ for the structure SiO_2_/IDT/LiNbO_3_/diamond/Si. (**a**) Rayleigh mode; (**b**) Sezawa mode.

**Figure 12:**
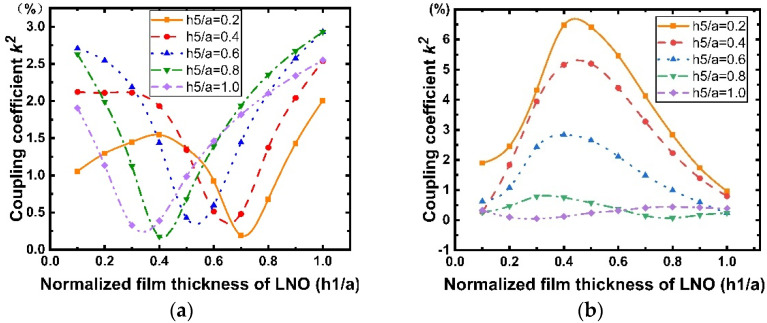
Dispersion curves of the Rayleigh and Sezawa waves electromechanical coupling coefficient ($k^{2}$) calculated for various values of $h_{1}/a$ for the structure SiO_2_/IDT/LiNbO_3_/diamond/Si. (**a**) Rayleigh mode; (**b**) Sezawa mode.

Figure 11 shows the dispersion curves of the phase velocity calculated for the Rayleigh and Sezawa modes with $h_{1}$ when the values of $h_{5}/a$ are 0.2, 0.4, 0.6, 0.8, and 1. According to Figure 11a,b, we can observe that the velocities of the Rayleigh and Sezawa waves decrease as $h_{1}$ and $h_{5}$ increase. This behavior is explained by the fact that the elastic velocity in the structure is close to that of the diamond substrate when the LiNbO_3_ and SiO_2_ layers are very thin, and these values decrease to reach those of the piezoelectric film. In the Sezawa mode, a high $v_{p}$ of 11.59 km/s could be obtained at $h_{1}/a\;$= 0.1 in the Sezawa mode; as $f_{0}=v_{p}/2p$, a higher $v_{p}$ will result in a higher frequency, which is beneficial for high-frequency device fabrication and performance. Figure 12 shows the calculated LiNbO_3_ thickness dependence of $k^{2}$. It is interesting that, in the Rayleigh wave mode (Figure 12a), $k^{2}$ shows the opposite trend and decreases dramatically compared with that for the structure without SiO_2_. In contrast, the Sezawa mode (Figure 12b) presents the same trend as before, and the value of $k^{2}$ increases significantly when the SiO_2_ thickness is small. In particular, when $h_{1}/a$ = 0.4, the maximum coupling coefficient of this mode is 6.47%.

#### 3.2.3. Effects of Temperature Compensation Layer Thickness on SAW Propagation Characteristics

**Figure 13:**
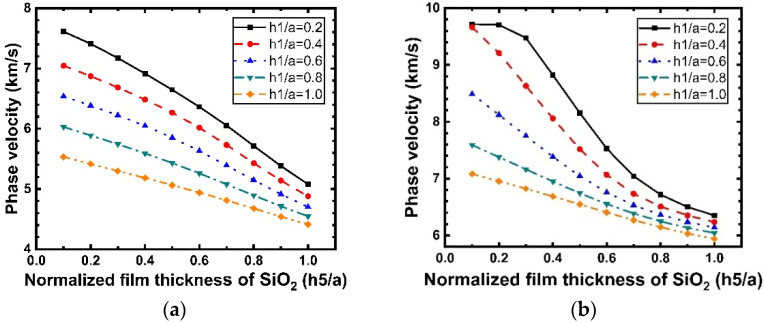
Dispersion curves of the Rayleigh and Sezawa waves velocity calculated for various values of $h_{5}/a$ for the structure SiO_2_/IDT/ LiNbO_3_/diamond/Si. (**a**) Rayleigh mode; (**b**) Sezawa mode.

**Figure 14:**
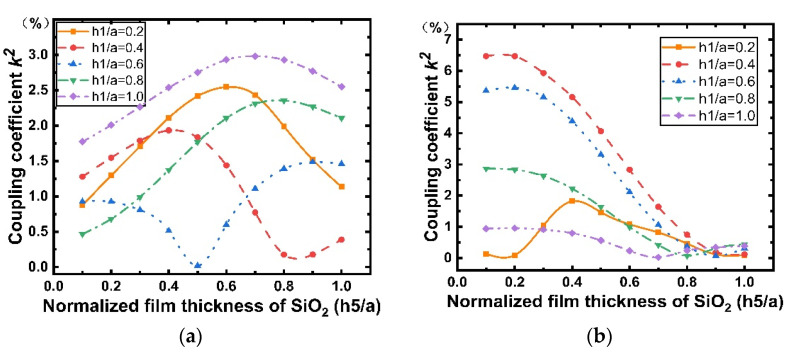
Dispersion curves of the Rayleigh and Sezawa waves electromechanical coupling coefficient ($k^{2}$) calculated for various values of $h_{5}/a$ for the structure SiO_2_/IDT/LiNbO_3_/diamond/Si. (**a**) Rayleigh mode; (**b**) Sezawa mode.

To study the implications of SiO_2_ on SAW propagation, different thicknesses of SiO_2_ were considered, and the results are shown in Figure 13 and Figure 14. Similarly, five different LiNbO_3_ thicknesses were used for comparison. From Figure 13, we can observe that although the thickness of the piezoelectric layer differs, the phase velocities of the Rayleigh wave and Sezawa wave in the five cases all decrease with the increase in the thickness of the SiO_2_ cover layer, where the SAW velocity slows down with increasing thickness of the SiO_2_ films because the SAW velocity in SiO_2_ is smaller than that in the IDT/LiNbO_3_/diamond/Si structure and the acoustic energy starts confining more in the SiO_2_ layer. In addition, according to Figure 14, when the thickness of the SiO_2_ layer is very small and increases, $k^{2}$ of the Sezawa wave increases and reaches the maximum (6.47%) at $h_{5}/a=0.2$. In contrast, in Figure 14a, it is observed that $k^{2}$ of the Rayleigh wave mode is generally lower than that of the Sezawa one but is still much higher than $k^{2}=1.2\%$ of the AlN/diamond structure [26]. Therefore, we can achieve a maximum value of $k^{2}$ by varying the thickness of the SiO_2_ layer deposited on the electrode. As expected, the SiO_2_ layer deposited in the electrode gap increases the electrostatic capacity of the device, thus reducing the maximum $k^{2}$ of the device without the SiO_2_ layer.

**Figure 15:**
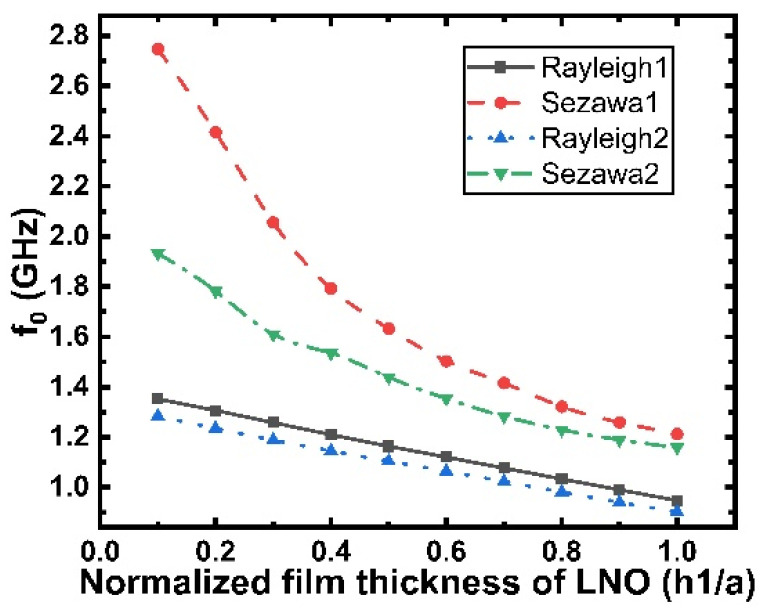
Plot of central frequency versus normalized LiNbO_3_ thickness ($h_{1}/a$). Rayleigh1, Sezawa1, and Rayleigh2, Sezawa2 are the two SAW modes before and after the layer thickness $h_{5}/a$ = 0.1 is added, respectively.

Figure 15 shows the central frequency ($f_{0}$) dependence on $h_{1}/a$ for the Rayleigh and Sezawa modes. It is clear that the center frequency of Sezawa mode is greatly affected when the SiO_2_ layer is covered, and correspondingly, the center frequency of Rayleigh mode is less affected. For both wave modes, the acoustic wave central frequency reduces with the increasing LiNbO_3_ layer thickness. However, within this range, the Rayleigh and Sezawa modes can still maintain high frequencies above GHz, so the layered structure still has a great advantage. These characteristics make this material system superior for high-frequency wideband applications.

The influence of temperature on the SAW devices has two main aspects: one is that thermal expansion of the SAW device structure causes the resonance frequency to change; additionally, temperature changes the material parameters. Under the influence of temperature, the material parameters in Equations (1)–(4) differ, where $c_{ijkl}$ becomes $c_{ijkl}\left(T\right),$ and ρ becomes ρ(T). Here, only the first-order temperature coefficient is considered.

(9) $$c_{ijkl}\left(T\right)=c_{ijkl}\left(T_{0}\right)\left[1+T_{c_{ijkl}}\left(T-T_{0}\right)\right]$$

(10) $$\rho \left(T\right)=\rho \left(T_{0}\right)\left[1+T_{\rho }\left(T-T_{0}\right)\right]$$

**Figure 16:**
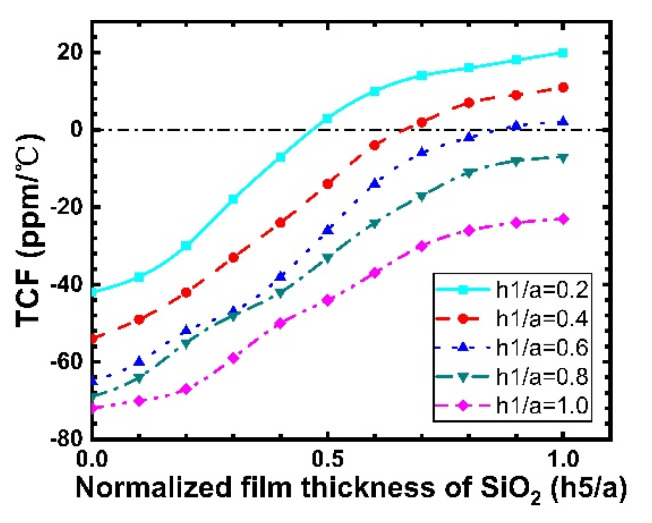
Calculated TCF changes versus SiO_2_ thickness.

The change in material parameters leads to a change in the SAW velocity, and the TCF is obtained using Equation (8). Figure 16 shows the TCF of a Rayleigh wave propagating along SiO_2_/IDT/LiNbO_3_/diamond/Si versus the normalized thickness of the SiO_2_ layer with different thicknesses of the LiNbO_3_ layer. For $h_{1}/a=$ 0.8 and 1, a negative value of TCF is observed for all values of SiO_2_ layer thickness. In the case of $h_{1}/a=0.2$, 0.4, and 0.6, a positive value occurs for a large $h_{1}$, and a TCF of zero is obtained. Note that the TCF of LiNbO_3_ single crystal reaches −75 ppm/°C, and it is apparent that the existence of the SiO_2_ film caused an improvement in the TCF. Usually, as shown in Figure 16, for a better TCF, a thicker SiO_2_ overcoat is preferred.

Therefore, the multilayer structure with added high-speed substrate can greatly increase not only the propagation velocity of SAWs but also the electromechanical coupling coefficient. A faster propagation velocity and larger $k^{2}$ correspond to a higher working frequency and larger bandwidth, which are more conducive to the design and manufacture of high-frequency wideband SAW devices. These results show that the TCF, working frequency, and bandwidth characteristics of SAW filters can be improved using a multilayer structure.

### 3.3. Transient and S Parameter Analysis

**Figure 17:**
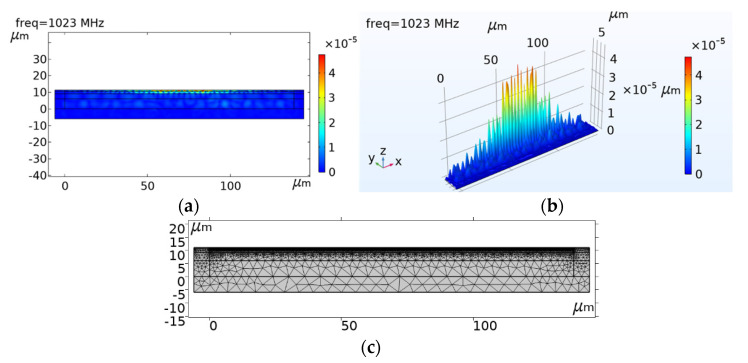
Simulation diagram of the total surface displacement of the model. (**a**) is a two-dimensional result and (**b**) is a three-dimensional result; (**c**) is the mesh density profile of complete structure, quadrilateral elements = 14319.

To simulate the real operation conditions of the SAW filter, the structure was analyzed in the time domain. Moreover, to reduce computational time, we modeled the SAW filter as a 2D system. In this model, $h_{1}/a$, $h_{4}/a,$ and $h_{5}/a$ were set to 0.7, 0.1, and 0.2, respectively. In this case, the eigenfrequency of the periodic model is $f_{0}=1023$ MHz, and the corresponding speed of the Rayleigh wave is $v_{p}=\lambda f$ = 6138 m/s. Figure 17 shows the total deformation of the system at the eigenfrequency of the simulation. The results indicate that the layered structure can lead to a confinement of the acoustic energy in the surface multilayer area. Further, this multilayered structure suppresses the bulk wave radiation and enables SAW energy confinement in the surface area, which is expected to improve the Q-factor.

**Figure 18:**
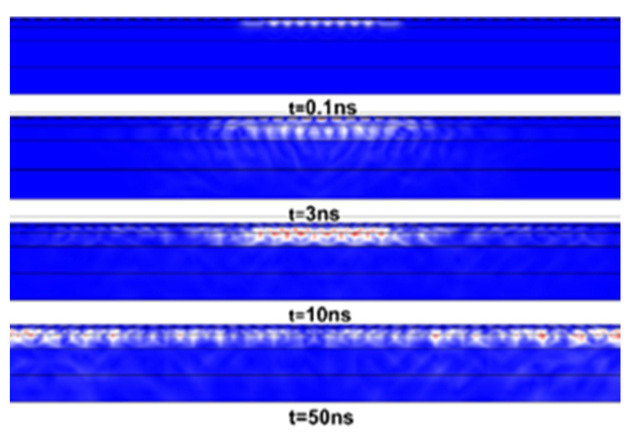
Simulation diagram of surface acoustic wave propagation in the structure.

The transient simulation was run for 50 ns with the timestep of 0.1 ns. Figure 18 shows the propagation characteristics of SAWs when the loading of the SAW filter is the harmonic electric voltage on the input IDT with an amplitude of 10 V and frequency equal to the eigenfrequency computed in the modal analysis, $f_{0}=1023$ MHz. As shown in Figure 18, the waves propagate on the surface of the filter, but some of them also propagate into the substrate, and at approximately *t* = 3 ns, the SAWs reach the output electrode.

**Figure 19:**
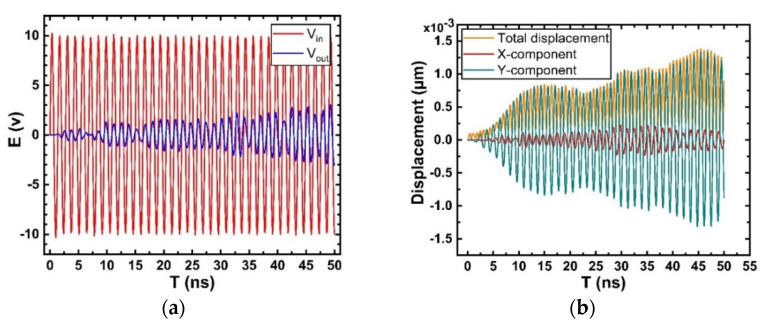
Results of transient analysis. (**a**) Input and output voltage on IDT; (**b**) Simulation diagram of the total surface displacement of the model and its X-Y component displacement.

Figure 19a shows input and output voltage on IDT as a function of time. The input signal is harmonic from start of simulation, but the output IDT needs some time to receive Rayleigh waves. The result is consistent with Figure 18.

Figure 19b shows the total displacement of the system and its X-Y component displacement changes in the time-domain simulation. The displacement direction of the Rayleigh wave has two components that are perpendicular and parallel to the surface. The particle vibration on the upper surface takes an elliptical path with two parts. It can be seen from Figure 19b that after a delay of 2 ns, the output begins to exhibit a significant vibration displacement. The displacement in the X direction lags slightly behind that in the Y direction, and the amplitude of the Y component is higher than that of the X component. This is because the crystal structure in the infinite base direction vibrates easier than that in the restricted direction, and the amplitude of the particle in the Y direction is greater than that in the X direction.

**Figure 20:**
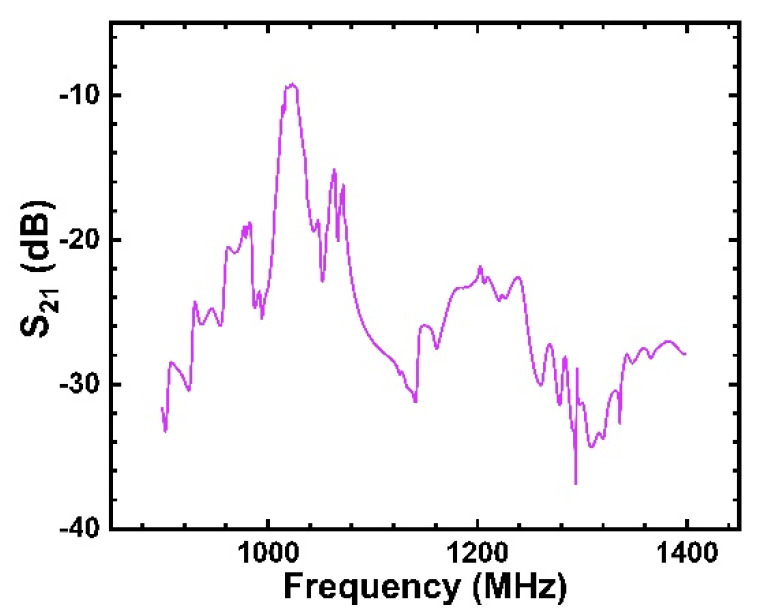
S_21_ characteristics of SAW filter of SiO_2_/IDT/LiNbO_3_/diamond/Si structure with Rayleigh mode.

Figure 20 shows the S_21_ characteristics in the frequency domain analysis of the filter designed with the SiO_2_/IDT/LiNbO_3_/diamond/Si structure. It can be seen that the transmission characteristics of the device are the best at the center frequency $f_{0}=1023$ MHz, and the minimum insertion loss is less than 10 dB. However, for the low-loss filter, there was still a large insertion loss. This is because this design parameter cannot obtain the optimal $k^{2}$, which leads to excessive insertion loss, and the insertion loss can be further reduced using the geometric result of a high $k^{2}$.

SAW parameters of SiO_2_/IDT/LiNbO_3_/diamond/Si compared with other SAW material systems are listed in Table 4. 

In contrast, SiO_2_/LiNbO_3_/diamond/Si is found to have a zero-temperature coefficient, and thus this material can be regarded as an ideal system for high-frequency wide-bandwidth applications. It is worth noting that in most of the literature, the width of the interdigital electrode reaches the submicron level; we set *λ* as 6 μm, and hence, the difficulty of manufacturing is reduced. If we continue to reduce its width, the operating frequency (*f*_0_) will double. For example, when *λ* = 1 μm, *f*_0_ exceeds 10 GHz in the Sezawa mode.

## 4. Conclusions

In this study, a new SiO_2_/IDT/LiNbO_3_/diamond/Si composite substrate structure designed to realize high-performance SAW devices was investigated. The effects of the thicknesses of IDT, LiNbO_3_, and SiO_2_ thin films on the structural performance were numerically evaluated using a 2D finite element model. In addition, the phase velocities, coupling coefficients, and temperature coefficients of the Rayleigh and Sezawa waves were calculated.

As a result, the layered structure was found to have a high phase velocity of up to 10 km/s and a high electromechanical coupling coefficient of 6.47% with a small temperature coefficient as low as 0 ppm/°C. Compared with those of traditional LiNbO_3_ single crystals, the phase velocity and electromechanical coupling coefficient of the layered structure increased by 144% and 17.7%, respectively. Furthermore, the layered structure has high temperature stability. From the results, we can conclude that the structure is promising for the realization of high-performance SAW filters at frequencies above the GHz range as long as the width of the interdigital electrode can be set reasonably. In the same way, the results provided in this study are of great importance for the future design of high-performance SAW devices on SiO_2_/IDT/LiNbO_3_/diamond/Si layered structures.

## Figures and Tables

**Table 1:** Geometric parameters for design.

Unit Element	λ (μm)	p (μm)	a (μm)	h1/a	h2 (μm)	h3 (μm)	h4/a	h5/a
value	6	3	1.5	0.1–1	3	6	0.1–0.5	0.1–1
**LCRF**	λ (μm)	p (μm)	a (μm)	***W***	***N_G_***	***N_I_***	***N_OUT_***	
value	6	3	1.5	20 λ	12	5	4	

**Table 2:** Material constants used in the simulations.

Item	Symbol	LiNbO_3_	Diamond	Si	Al	SiO_2_
Density (Kg/m^3^)	ρ	4700	3515	2329	2695	2200
elasticity matrix (GPa)	$c_{11}$	203	1158	166	111	78.5
	$c_{12}$	70.72	85	64	61	16.1
	$c_{13}$	57.9	85	64	61	16.1
	$c_{33}$	222.1	1158	166	111	78.5
	$c_{44}$	75.8	537	79.5	25	31.2
piezoelectric constant (C/m^2^)	$e_{15}$	4.455	-	-	-	
	$e_{31}$	1.692	-	-	-	
	$e_{33}$	2.322	-	-	-	
dielectric constant (10^−11^ F/m)	$\varepsilon_{11}$	43.6	-	11.8	-	3.32
	$\varepsilon_{33}$	34.65	-	11.8	-	3.32
Temperature coefficients of elastic constants (10^−4^/°C)	$Tc_{11}$	−1.74	−0.14	−0.53	−5.9	2.39
	$Tc_{13}$	−1.59	−0.57	−0.75	−0.8	5.84
	$Tc_{33}$	−1.53	−0.14	−0.53	−5.9	2.39
	$Tc_{44}$	−2.04	−0.125	−0.42	−5.2	1.51
Temperature coefficients of mass density (10^−6^/°C)	$T_{\rho }$	-	−3.6	-	−1.65	−54

**Table 3:** Boundary conditions of the model.

Boundary	Mechanical Boundary Conditions	Electrical Boundary Conditions
Γ6, Γ7	Free boundary	Potential/Grounding
Γ1, Γ2, Γ3, Γ5	Free boundary	Continuity
Γ4	Fixed constraint	Grounding
ΓL, ΓR	Periodic conditions	

**Table 4:** SAW parameters of SiO_2/_LiNbO_3_/diamond/Si compared with other material systems.

Ref	Substrate	f_0_ (GHz)	Vp (km/s)	$k^{2}$	TCF (ppm/°C)
[27]	LiNbO_3_/SiO_2_/SiC	1.28	2.78	22%	−63.8
[28]	LiNbO_3_/SiO_2_/Si	1.28	3.5	25%	−57.7
[26]	AlN/diamond	10	10	0.2%	/
[29]	TeO_3_/SiC/LiNbO_3_	/	4.39	9.7%	TCD:0–60
[30]	LiNbO_3_/diamo-nd/Si	/	12.4	4.8%	/
This work	SiO_2_/IDT/LiNbO_3_/diamond/Si	≥1	≥9	6.5%	0

## Data Availability

Not applicable.
